# Population Genomic Sequencing Delineates Global Landscape of Copy Number Variations that Drive Domestication and Breed Formation of in Chicken

**DOI:** 10.3389/fgene.2022.830393

**Published:** 2022-03-22

**Authors:** Xia Chen, Xue Bai, Huagui Liu, Binbin Zhao, Zhixun Yan, Yali Hou, Qin Chu

**Affiliations:** ^1^ Institute of Animal Husbandry and Veterinary Medicine, Beijing Academy of Agriculture and Forestry Sciences, Beijing, China; ^2^ CAS Key Laboratory of Genomic and Precision Medicine, Beijing Institute of Genomics, Chinese Academy of Sciences, Beijing, China; ^3^ China National Center for Bioinformation, Beijing, China; ^4^ College of Life Sciences, University of Chinese Academy of Sciences, Beijing, China

**Keywords:** chicken, copy number variation, evolution, domestication, breed-specific

## Abstract

Copy number variation (CNV) is an important genetic mechanism that drives evolution and generates new phenotypic variations. To explore the impact of CNV on chicken domestication and breed shaping, the whole-genome CNVs were detected via multiple methods. Using the whole-genome sequencing data from 51 individuals, corresponding to six domestic breeds and wild red jungle fowl (RJF), we determined 19,329 duplications and 98,736 deletions, which covered 11,123 copy number variation regions (CNVRs) and 2,636 protein-coding genes. The principal component analysis (PCA) showed that these individuals could be divided into four populations according to their domestication and selection purpose. Seventy-two highly duplicated CNVRs were detected across all individuals, revealing pivotal roles of nervous system (*NRG3*, *NCAM2*), sensory (*OR*), and follicle development (*VTG2*) in chicken genome. When contrasting the CNVs of domestic breeds to those of RJFs, 235 CNVRs harboring 255 protein-coding genes, which were predominantly involved in pathways of nervous, immunity, and reproductive system development, were discovered. In breed-specific CNVRs, some valuable genes were identified, including *HOXB7* for beard trait in Beijing You chicken; *EDN3*, *SLMO2*, *TUBB1*, and *GFPT1* for melanin deposition in Silkie chicken; and *SORCS2* for aggressiveness in Luxi Game fowl. Moreover, *CSMD1* and *NTRK3* with high duplications found exclusively in White Leghorn chicken, and *POLR3H*, *MCM9*, *DOCK3*, and *AKR1B1L* found in Recessive White Rock chicken may contribute to high egg production and fast-growing traits, respectively. The candidate genes of breed characteristics are valuable resources for further studies on phenotypic variation and the artificial breeding of chickens.

## Introduction

Since the days of Darwin, it has been recognized that a succession of livestock species leads to significant differences in behavior, morphology, and physiology in response to domestication compared with their wild ancestors (Charles Darwin, 1859). Deciphering the genetic basis, molecular mechanisms, and evolutionary driving forces of the complex traits that have been innovated or reshaped by human manipulation during livestock domestication and breed formation has long captured the interest of animal biologists in resource utilization and animal breeding. The development of genomic sequencing technologies provides more powerful tools for a comprehensive understanding of the genetic architectures and evolutionary trajectories of complicated traits in humans and animals (International Chicken Genome Sequencing, 2004; Scally et al., 2012). In addition to the single nucleotide polymorphisms (SNPs), structural variation has been recognized as another crucial factor for driving phenotypic variations, complex diseases, and developmental abnormalities, such as obesity, diabetes, psychiatric diseases, and cancers (Sebat et al., 2004; Feuk et al., 2006; Freeman et al., 2006; Redon et al., 2006). Copy number variation (CNV) is a major type of structural variation in the genome and generally defined by the insertion, duplication, or deletion of a relatively large size of DNA with length >50 bp; this process contributes to much more variability than SNPs (Iafrate et al., 2004; Freeman et al., 2006). Various molecular mechanisms have been proposed for the formation of CNVs, with non-allelic homologous recombination being regarded as a major source of structural variation in regions of extended homology (Hastings et al., 2009; Arlt et al., 2012). CNVs typically affect gene expression and phenotypic specialization directly through dosage compensation (Zhou et al., 2011), or indirectly through altering gene expression by reshaping the three-dimensional genome architecture and local DNA accessibility; thus, they influence the regulatory relationships or intensities between the regulatory elements and targeted genes (Beroukhim et al., 2016; Franke et al., 2016; Rice and Mclysaght, 2017).

CNVs play an important role in the evolutionary adaptation of an organism under both natural and artificial selection, affecting fitness and reproductive ability, which indicates a significant source of adaptive potential. The copy number of *AMY1* is strongly correlated with the evolution of diets; specifically, individuals with high-starch diets have more *AMY1* copies than those with low-starch diets (Perry et al., 2007). Minias et al. (2019) reported that the evolution of the *MHC* copy number in birds was driven by different selective pressures, such as by intra- and extracellular pathogens and parasites. Besides, the CNVs have been implicated in phenotypic variability of traits important for domestication and breed formation in many livestock species. The duplication of *KIT* is significantly associated with white coat color in pigs (Giuffra et al., 2002) and cattle (Durkin et al., 2012). The chicken pea-comb is caused by the duplication of *SOX5* in intron 1 (Wright et al., 2009). The CNV of *ZNF280AY* was negatively correlated with male reproduction trait in Holstein and Simmental bulls (Pei et al., 2019).

Domestic chickens (*Gallus gallus domesticus*), which were initially domesticated from the red jungle fowl (RJF) subspecies *Gallus gallus spadiceus* in East Asia (Peters et al., 2016; Wang M.-S. et al., 2020), have undergone climate and environmental changes, artificial domestication, and commercial breeding for a long period. After thousands of years of domestication and selection, domestic chickens have been separated into several hundreds of distinct breeds spreading globally, and their appearance, behavior, growth, and reproduction traits vary from breed to breed. Hence, chickens are an excellent model to explore the evolution and domestication of animals and to identify the evolutionary spectrum of CNVs controlling domestication, breed formation, and important economic traits. However, most researches mainly focused on yielding CNV maps for chickens (Crooijmans et al., 2013; Tian et al., 2013; Yi et al., 2014; Sohrabi et al., 2018) or identifying CNVs based on some certain traits (Zhang et al., 2014; Xu et al., 2017). The evolutionary spectrum of CNVs (e.g., magnitudes, trajectories, and mechanisms) affecting important economic traits of livestock species during their domestication and breed formation remains largely elusive. Therefore, the main aim of the study is not merely yielding an exhaustive CNV map for chicken but also exploring the role of CNVs in evolution and domestication. What’s more, the exclusive CNVs of each breed were systematically analyzed to identify the genes contributing to breed-specific characteristics. This study offers a new perspective on the evolutionary spectrum of CNVs under artificial selection during chicken domestication and breed shaping, and the results may help reveal potential genetic mechanisms for some meaningful traits in chicken, accelerate breeding programs, and improve the quality and efficiency of production.

## Materials and Methods

### Sample Preparation and Sequencing

We collected 47 samples from 6 typical breeds, including 4 Chinese indigenous breeds [eight Xinghua (XH) chickens from Guangdong province, eight Luxi Game (LXG) fowl from Shandong province, eight Beijing You (YOU) chickens from Beijing, nine Silkie (SILK) chickens from Jiangxi province], and 2 introduced commercial breeds [eight Recessive White Rock (RW) chickens, six White Leghorn (WL) chickens]. SILK chicken is known for its fluffy plumage, dark blue bones and skin, and five toes on each foot. Besides, the SILK chicken is thought to have medicinal properties. LXG chicken is a famous gamecock breed. YOU chicken is a dual-purpose breed with a special appearance (crest on the head, beard under the lower jaw, feathers on both shanks, and five or more toes on each foot). XH chicken has slow growth, low production, and favorable meat quality. As for the two commercial breeds, WL chicken is famous for high egg production and RW chicken for high meat yield. Samples were randomly collected to avoid genetic affinity. Blood samples from 47 birds were used to construct libraries and were sequenced using Hiseq2000 platform. About 95–117 million clean paired-end reads with a length of 100 bp were produced for each sample. The raw sequence data reported in this paper have been deposited in the Genome Sequence Archive in the BIG Data Center, Beijing Institute of Genomics, Chinese Academy of Sciences, under the accession number PRJCA000093, and they are publicly accessible at http://bigd.big.ac.cn/gsa. Data of four wild chickens, Red Jungle Fowl (RJF), were downloaded from the Sequence Read Archive (SRA) database with accession number PRJNA241474 (Wang et al., 2015). In total, 51 individuals from 7 breeds were included for whole-genome analysis (Supplementary Table S1).

### Profiling of Whole-Genome CNVs

We analyzed the whole-genome CNVs using four programs, namely mrFAST, CNVnator, BreakDancer, and Pindel, which have disparate algorithms. mrFAST algorithm is mainly used to identify duplicated segments and simultaneously predict absolute CNVs for the duplicated segments (Alkan et al., 2009). CNVnator is based on the read depth signals, representing greater challenges when calling retrotransposons, duplications, and balanced CNVs (Abyzov et al., 2011). CNVnator is able to discover CNVs in a vast range of sizes, from a few hundred bases to mega bases in length, in the whole genome. BreakDancer is a bioinformatics tool that relates paired-end read alignments from a test genome to the reference genome for the purpose of comprehensively and accurately detecting various types of structural variation including deletion, insertion, inversion, intra-chromosomal translocation, and inter-chromosomal translocation. While BreakDancer is not suitable for detecting small variants. Pindel is a split read-based pattern growth approach to detect CNVs. Pindel performs better in detecting small insertions and deletions and can only detect a limited number of large structural variations (> 1 kb) (Abyzov et al., 2011; Liu et al., 2020).

First, we used mrFAST and mrCaNaVaR to exploit the whole-genome CNVs. The reference genome was first masked for repeat elements using RepeatMasker (Smit and Green, 2013) and Tandem Repeats Finder (Benson, 1999). mrFAST was implemented to align paired-end reads to the reference genome in a single mode, accommodating five edit distances, which reported all possible aligned locations for each read. mrCaNaVaR operated the whole-genome shotgun sequence algorithm, which utilized three categories of sliding windows to calculate the normalized read depth, identify large segmental duplications and deletions, and calibrate their absolute copy numbers. The raw read depth in each window was normalized based on its GC content via a LOESS-based smoothing technique. Segmental duplication and deletion regions were declared where at least six out of seven consecutive 5-kb non-masked windows with 1 kb sliding steps presented significantly increased and decreased read depths (mean ± 4*standard deviation); their boundaries were subsequently refined by 1 kb non-masked windows having increased and attenuated read depths (mean ± 2*standard deviation). The absolute copy numbers within 1 kb of non-overlapping non-masked windows were predicted based on the normalized read depths.

For CNVnator, BreakDancer, and Pindel, the clean paired-end reads were aligned to the indexed chicken reference genome (galGal4) by performing the maximal exact match algorithm in BWA (Li and Durbin, 2009), where reads were either uniquely mapped or randomly located at a place if they had multiple alignments. After alignment, we exploited BreakDancerMax to first classify the aligned read pairs into six types: normal, deletion, insertion, inversion, intra-chromosomal translocation, and inter-chromosomal translocation based on the separation distances and alignment orientation between the paired reads. Phred-style quality scores were required greater than 30, with sufficient mapping quality, and a confidence score of 90 was required. Only the deletion results were retained for further analysis. Pindel with split-reads approach was used with default parameters except that the cut off value for the number of supporting reads was set to 5. The deletion with length ≥50 bp was selected.

For CNVnator, the read depth signals were calculated as the count of mapped reads within consecutive non-overlapping bins and further corrected according to the corresponding GC content. A mean-shift technology was applied to partition these read depth signals into segments with presumably different underlying copy numbers. Based on these, the putative CNVs were further predicted by performing statistical significance tests, whose exact copy numbers were estimated as normalized read depths. Since the optimal bin size is crucial for CNV calling, it was once recommended as the one at which the ratio of the average read depth signal to its standard deviation is approximately 4–5 (Abyzov et al., 2011). The bin size in our study was determined as 500 bp. In order to avoid false discovery issues, we subsequently filtered out the CNVs with q0 >50% (zero mapping quality). And only the deletion results were retained for further analysis. We finally prepared the CNV dataset using mrFAST duplication result as duplication regions and integrating the deletion results supported by at least two of four softwares as deletion regions.

### Population Genetics Analysis

We integrated the CNV into copy number variation region (CNVR) based on 1 bp overlap. If a CNVR was present in the sample, we labeled 1 for the region, and 0 for absent event. For example: sample 1 has a deletion of chr1:50500-51499, sample 2 has a deletion of chr1:50600- 52199, sample 3 has a deletion of chr1:53000-53999. Then the CNVR is chr1:50500-52199 and chr1:53000-53999. For the first CNVR, sample 1 and 2 present, but sample 3 does not. Then we performed principal component analysis (PCA) using princomp () under R environment (Team, 2021).

To explore the CNVR that shows stratification among populations, we calculated Vst among pairwise populations (Redon et al., 2006). We used a copy number of 1 kb repeat-free non-overlapping windows provided by mrFAST in CNVR. Vst was calculated by the following formula:

$$Vst=\frac{\sigma_{T}^{2}-\frac{n_{1}\times \sigma_{1}^{2}+n_{2}\times \sigma_{2}^{2}}{n_{1}+n_{2}}}{\sigma_{T}^{2}}$$

where $\sigma_{T}^{2}$ is the total variance among all unrelated individuals, $\;\sigma_{1}^{2}$ and $\sigma_{2}^{2}$ are the variance within populations 1 and 2, respectively, and $n_{1}$ and $n_{2}$ are the population sizes.

The Vst value fluctuated between 0 and 1. The region where Vst = 0 exhibits no differentiation, whereas Vst = 1 means complete differentiation. When comparing one population with the other, if all the Vst values were above 0.4, we selected the region as a candidate breed-specific region.

### Gene Annotation and Enrichment Analysis

We downloaded gene annotations from the Ensembl database with version 78. If there was overlap of at least 1 bp with CNV, the gene was annotated as CNV associated gene. We performed GO and KEGG enrichment analyses using DAVID (https://david.ncifcrf.gov/). The ingenuity pathway analysis (IPA, http://www.ingenuity.com) software was used to analyze the enrichment based on the IPA knowledgebase. Categories of disease and disorder, molecular and cellular functions, and physiological system development and functions are developed in IPA. The *p*-value was calculated by right-tailed Fisher’s exact tests.

### Different Expression Analysis

Nine RNA sequencing data of cerebrum samples from LXG, SILK, RW, and WL male chickens (two for LXG, RW, and WL respectively and three for SILK) (Hou et al., 2020), as well as two transcriptomes of cerebrum samples from RJFs (one male and one female) with accession numbers SRR306710-306711 (Brawand et al., 2011) were downloaded and analyzed together. The clean sequencing data were mapped to the reference genome (galGal4) by TopHat v2.0.13 (Trapnell et al., 2009). The different expression level for the comparison of RJF versus all domesticated chickens was detected by HTSeq and DESeq with filters of Padj <0.01 and fold change (FC) >1.5 (Anders and Huber, 2010). We obtained the average expression level of differentially expressed genes (DEGs) for each breed based on the normalized count value provided by DESeq, and then we calculated the Pearson correlation coefficient with the average copy number of each breed.

## Results

### Identification of Whole-Genome Copy Number Variation

In this study, 47 birds from four Chinese indigenous breeds and two introduced commercial breeds were randomly selected and whole-genome sequenced (Supplementary Table S1). SILK, YOU, LXG, and XH chickens were sampled from Jiangxi, Beijing, Shandong, and Guangdong provinces, respectively. The two commercial breeds were RW and WL, representative of broiler and layer, respectively. Additionally, the genome sequence of four RJFs, which represented the wild chicken, was downloaded from the SRA database (Wang et al., 2015). Herein, whole-genome sequencing data from 51 individuals for seven breeds were included in our analysis. The sequencing data were endowed with an approximate coverage depth of 10× paired-end reads per individual and only autosome data were used (Supplementary Table S2).

To complementarily capture disparate aspects of CNVs, we applied multiple approaches that simultaneously characterize distinct properties of CNVs based on different principles. For duplication, the mrFAST method was performed, and an average of 379 duplications for each individual was detected. For deletion, we integrated four methods of mrFAST, CNVnator, BreakDancer, and Pindel, and short variations (< 50 bp) were removed. The average deletion numbers for each bird detected by four different methods were 54, 794, 2,282, and 2,278, respectively (Supplementary Table S2). Approximately 35.61% of these deletion events were discovered *via* at least two methods, and only 0.26% were shared by all four methods. For next analysis, only deletions supported by at least two methods were retained, with an average of 1,936 deletions per individual.

Then, we merged the above results of 19,329 duplications and 98,736 deletions to obtain CNVRs. A total of 11,123 CNVRs (length range: 68–2,802,702 bp) were elaborately identified, which represented 7% of the autosome genome, with the mean and median length of 6,748 bp and 986 bp, respectively. Most CNVRs (8,834) were denominated as “deletion,” and 1,911 were “duplication,” while the other 378 were “complex,” which included both “deletion” and “duplication” events (Supplementary Table S3). From the viewpoint of frequency spectrum that shared among CNVRs of the 51 individuals, we found 4,515 singletons, 5,121 lowly shared CNVRs with frequencies less than 20% excluding singletons, 1,200 medially shared with a frequency of 20–80%, 135 highly shared with a frequency of 80–99%, and 152 common CNVRs, resulting in a typical skewed distribution (Supplementary Figure S1). Meanwhile, the CNVR lengths presented a typical skewed distribution identically with a higher proportion of smaller CNVRs (Supplementary Figure S1), generally indicating a selective genetic load on CNVs.

Furthermore, we compared the CNVRs with the Ensembl BioMart v78 database (galGal4) to identify genes contained in those CNVRs and filtered the singleton CNV in the population to ensure a high quality of functional CNVs. A total of 2,636 protein-coding genes overlapped with duplications and deletions in CNVRs. Then, Kyoto Encyclopedia of Genes and Genomics (KEGG) pathway and Gene Ontology (GO) term analysis were performed using the DAVID database. Genes with copy number variations were mainly associated with axon guidance, ATP binding, GTPase activator activity, and phosphatidylinositol binding based on the GO term, and they were involved in vascular smooth muscle contraction, oocyte meiosis, and gap junction according to KEGG pathway analysis (Supplementary Table S4). This suggests that the functional shape of CNVs mainly modifies behavior, energy, and reproduction, which are typical target traits in chicken domestication.

### Population Characteristics of Whole-Genome Copy Number Variation

**FIGURE 1:**
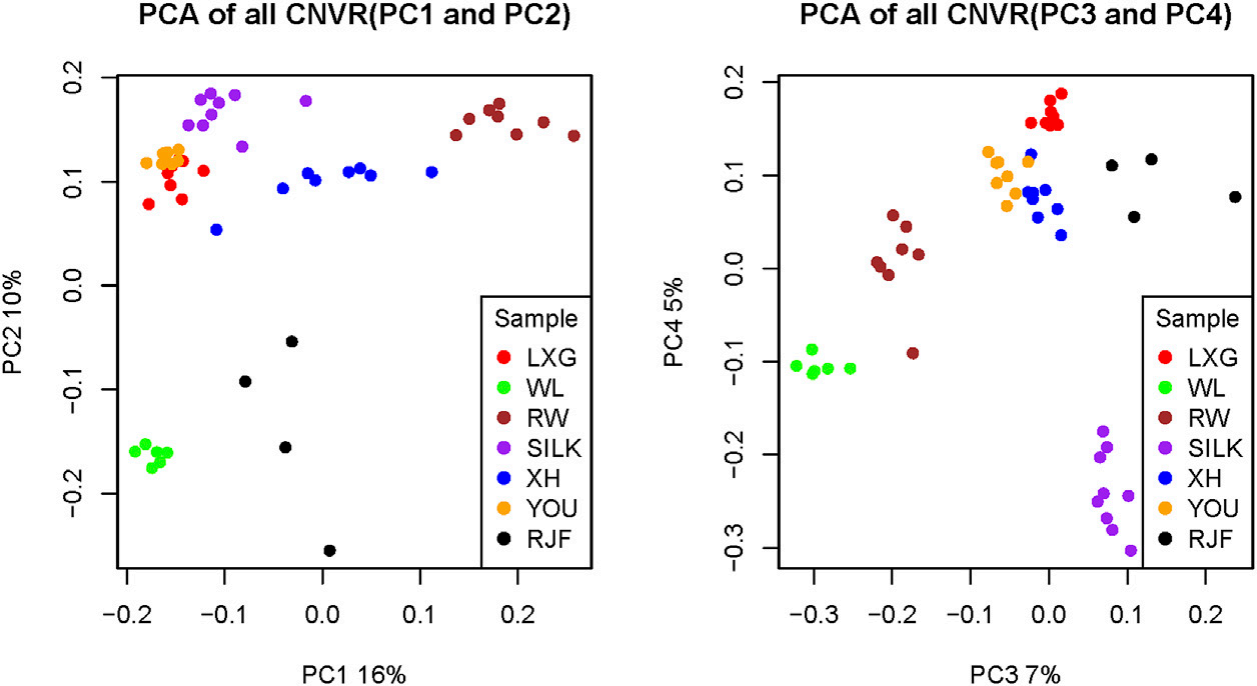
The principal component analysis (PCA) plot of all CNVRs. **(A)** Samples were divided into four groups: commercial layer, commercial broiler, RJFs, and Chinese native breeds. **(B)** Samples were divided more detailly that SILK was further separated from the other Chinese native breeds. XH: Xinghua; LXG: Luxi Game fowl; YOU: Beijing You; SILK: Silkie; RW: Recessive White Rock; WL: White Leghorn; RJF: Red jungle fowl.

PCA was performed on all CNVRs detected in the seven populations to explore the population stratification based on the variations (Figure 1). By integrating PC1 and PC2, which interpreted 16% and 10% of the phenotypic variance respectively, the samples were primarily divided into four clusters. The first and second clusters represented the introduced meat dynamo RW and the introduced layer dynamo WL, respectively. The third cluster was the wild RJF from Yunnan and Hainan provinces of China. The fourth was a cluster of Chinese native breeds, which could also be separated from each other, except for YOU and LXG breeds. Based on PC3 and PC4, which described 7% and 5% of the total variance, respectively, these populations could also be distinguished apparently, and SILK chicken was further separated from other Chinese native breeds. These findings suggested that the CNV spectrum can substantially reflect breed differentiation in light of genetic divergence and artificial selection.

### Ubiquitous Copy Number Variations in Chickens

The common copy number variations of chickens included in all the wild and domesticated individuals indicate that the genomic characteristics might be relevant to speciation of chicken or its ancestors. As mentioned, 152 CNVRs were commonly detected among all 51 individuals. Among these regions, 72 were highly represented duplication CNVRs and 172 corresponding genes were involved, with minimum and maximum copy numbers of 3 and 373, respectively. They were predominantly enrichment in feather keratin, sensory perception, metabolic, reproductive processes, muscle contraction, and immunity (Supplementary Table S5).

As expected, highly duplicated segments of keratin genes in terms of feather keratin and feather-keratin-like protein were observed in chromosomes 25 and 27, with copy numbers ranging from 3 to 90. *MUC5B*, a member of the mucin family, was discovered to be intensively duplicated with varied copy numbers ranging from 17 to 42. Additionally, four genes, *MRPS35*, *SMARCD3*, *KHDRBS2*, and *ANO3* exhibited a minimum copy number of more than 30 and a maximum of over 60. *NRG3* and *NCAM2*, which are associated with development and differentiation of nervous system, were also included, and their copy numbers ranged from 3 to 42 and 15 to 40, respectively. Besides, 3–5 copy numbers of *VTG2* and 3–6 of *GBE1* were identified.

### Copy Number Variations Correlated With Chicken Domestication

The loci with high copy number differentiation between wild and domestic populations are considered as candidates driving adaptive selection. Therefore, the copy number variations between RJF and all the six domestic breeds were compared to identify the potential genes associated with chicken domestication. The method of Vst was used to estimate the population differentiation of copy numbers (Redon et al., 2006). A total of 235 CNVRs containing 255 protein-coding genes were discovered in the windows with 1% toppest Vst (> 0.2042) (Supplementary Table S6). The gene enrichment analysis by IPA showed that these copy number differentiated genes are predominantly involved in nervous system development, reproductive system development and function, cellular growth and proliferation, and immunity (*p* < 0.01) (Supplementary Table S7).

First, quite a few of genes which were involved in the development and function of nervous system were found to be highly differentiated in gene copy, including *NRG3*, *BICD1*, *ANKRD11*, *SEMA3F*, *UNK*, *EPS15L1*, *KIF3C*, *FBXO41*, *MYT1*, *NR2E1*, *PSMC5*, *SEMA3F*, *SOX2*, *PTPRM*, and *RPS6KB1*. A CNVR in exon 1 of *NRG3* was observed to have an average copy number higher than 30 in RJFs, but the median copy number in domestic individuals was 16 (Supplementary Figure S2). Second, some genes associated with immunity or stress response, such as *CBFA2T3*, *IL22RA1*, *HERPUD1*, *ARIH2*, *GATA3*, *TNFAIP8L1*, *CD86*, *CD8A*, *SMARCD2*, and *TNPO3*, were also detected possessing differential copy numbers in genome. Besides, some other valuable genes correlated with energy metabolism, cell growth and proliferation or reproduction functions were also found, for instance, *GBE1*, *ACAT*, *ACO2*, *HEP21*, *INHA*, *GPC6*, *MEI1*, *PTPRM*, *RPS6KB1*, etc. *GBE1* presented one copy number more in intron 7 in RJF than in all the other six domesticated chicken breeds (Supplementary Figure S2).

### Breed-Specific Copy Number Variations

Considering that CNV may be correlated with the origin or breeding of different chicken breeds, we further compared each domestic breed with each of the other six breeds to determine the extremely specific regions. If the six Vst values of a region were over 0.4, it was considered as a breed-specific region. In total, 600 CNVRs harboring 90 annotated genes were found to be breed-specific (Supplementary Table S8).

RW chickens exhibited 64 breed-specific CNVRs and 39 protein-coding genes, showing the highest number of specific genes across all the six domestic breeds. Genes specific to RW chickens may be candidate genes for growth and muscle traits. For example, *POLR3H*, *MCM9*, *ASF1A*, *MYH1B*, and *DOCK3* may contribute to rapid growth and plump muscle traits. Furthermore, WL chickens possess 63 regions and 19 genes. Many genes, i.e., *GSK3B*, *CSMD1*, *NTRK3*, and *STRBP*, may be associated with reproduction traits.

**FIGURE 2:**
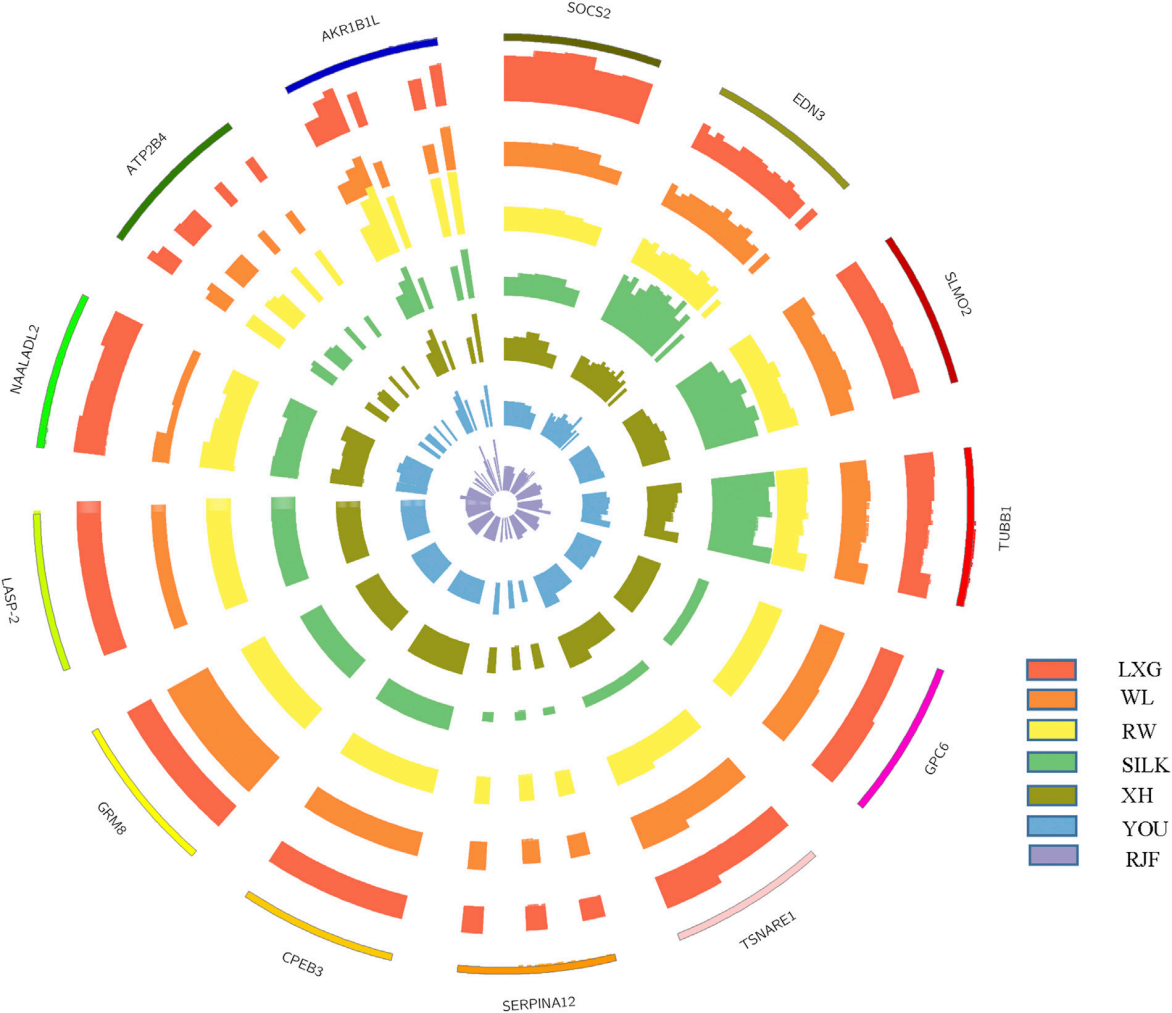
The schematization for a part of copy number variation genes distinguished in different chicken breeds. The outermost circle indicates the CNV-involved gene regions, with the thickness implying the normal diploid. The other circles from outside to inside indicate the breeds of Luxi Game fowl (LXG), White Leghorn (WL), Recessive White Rock (RW), Silkie (SILK), Xinghua (XH), Beijing You (YOU), and red jungle fowl (RJF) in turn, whose thickness proportionally means the estimated copy number.

For the four Chinese native chicken breeds, LXG, SILK, YOU, and XH, we detected 32, 23, 17, and 2 regions, corresponding to 8, 15, 7, and 1 protein-coding gene, respectively. From the schematization of copy numbers for a part of genes and the CNV-involved gene segments that distinguished chicken breeds (Figure 2), we could notice the difference clearly. In LXG chickens, *SOCS2* presented a copy number of about 4, which was significantly higher than that of the other breeds. *EDN3* had around 4 copies exclusively in SILK chickens, and its neighboring genes *SLMO2* and *TUBB1* also had higher copy numbers than other breeds, which have been frequently targeted as tetra- or penta-ploid (Han et al., 2014; Yi et al., 2014). Moreover, SILK chickens have specially experienced one-copy deletion on *GPC6*, *TSNARE1*, and *SERPINA12*. For XH chickens, *CPEB3* was the only detected breed-specific gene, showing one-copy higher particularly. It was reported that *CPEB3* was related to pathway of oocyte meiosis (Wang Y. et al., 2020).

### Correlation Between Copy Number Variation and Gene Expression in Cerebrum

Considering that variation in gene copy number is expected to change the RNA expression level, the DEGs between wild RJF and each of the other four domesticated breeds (LXG, SILK, RW, and WL) in cerebrum published by our team (Hou et al., 2020) were further explored. A total of 28 genes were shared among the domestication-related copy number differentiated genes. Additionally, the correlations between the gene expression and copy numbers were intersected (Supplementary Table S9). More than half (16/28) of the 28 overlaps presented a significant correlation between copy number and gene expression, with absolute Pearson’s correlation coefficients larger than 0.88 (*p* < 0.05). Among the significantly correlated genes, most (9/16) showed negative correlations.

As mentioned, 15 genes participating in the development and function of nervous system showed copy number variation specifically in RJF, among which seven (*BICD1*, *ANKRD11*, *SEMA3F, UNK*, *EPS15L1*, *KIF3C*, and *FBXO41*) were significantly differentially expressed in the cerebrum, with one (*ANKRD11*) upregulated and the other six downregulated in domesticated breeds compared with RJF. Meanwhile, except for *BICD1*, six showed negative correlation with copy number.

## Discussion

In this study, whole-genome CNVs were explored in RJF and six domestic chicken breeds. The particularity for each breed predominantly contributed to their inner divergences, for example, high egg performance for WL, high meat yield for RW, fluffy plumage, black skin, and medical purpose for SILK, game fighting for LXG, low production and favorable meat quality for XH, and special appearances for YOU chickens. By combining multiple methods, four for deletions and one for duplications, we systematically and exquisitely analyzed the copy number information in 51 individuals and obtained a comprehensive map of genomic variations for chickens. A total of 19,329 duplications and 98,736 deletions were detected, covering 11,123 CNVRs and 7% of the autosome genome.

PCA results with both duplication and deletion CNVRs revealed that the seven chicken populations were well divided into four parts according to the degree of artificial selection pressure and the purpose of domestication: commercial broiler, commercial layer, Chinese native breeds, and their ancestor RJF. These results were consistent with the population stratification pattern based on SNPs (Hou et al., 2020), revealing that CNV is a powerful and effective tool for distinguishing samples and that CNVs may also participate in the driving of chicken domestication.

### Genes Relating to Chicken Evolution

As an important genetic source, copy number variation can provide a good perspective for exploring the track of animal evolution. Therefore, 72 high duplication CNVRs (corresponding to 172 genes) detected in all individuals were analyzed to explore chicken evolution, which might be characterized as the genomic features specialized in chicken species.

As expected, feather keratin (*FK* or *F-KER*), olfactory receptor (*OR*), and neuregulin 3 (*NRG3*) were detected CNVs as previous studies reported. Keratin is a main protein that makes up avian feathers (Ramakrishnan et al., 2018). *FK* or *F-KER* was observed with intensive copy number duplication in current study, which is highly consistent with the results of Hillier et al. (2004). *OR* genes, which plays a pivotal role in the sense of smell among vertebrates, exhibited a copy number around 20 in this study. Zhou et al. (2019) reported more than 90 *OR* genes in chicken, which was still much fewer than in mammals like humans (396), dogs (857), cats (865), cattle (881), and pigs (1113) (Bu et al., 2019). The contraction of *OR* genes in chicken genome may be responsible for the relatively poor sense of smell for bird species. *NRG3* was found to be associated with the development and differentiation of nervous system. Abe et al. (2017) also detected copy number variations and frameshift deletion of *NRG3* in chicken.

Besides, some genes, such as *MUC5B*, *NCAM2*, and *VTG2,* were discovered with copy number differences in all individuals. *MUC5B* is a major contributor to the lubricating and viscoelastic properties of whole saliva, normal lung mucus, and cervical mucus (Roy et al., 2014; Käsdorf et al., 2017). The gene *NCAM2* has been proposed to contribute to neurodevelopmental disorders in humans (Winther et al., 2012). Furthermore, we identified that *VTG2*, which is the yolk precursor protein expressed in females of nearly all oviparous organisms, possessed 3–5 copies across all individuals. The presence of multiple copies of *VTG2* in the genome was due to the gene family expansion from common ancestor (Biscotti et al., 2018) and occurred prior to the most recent common ancestor of birds (Brawand et al., 2008; Finn et al., 2009).

### Genes Involved in Chicken Domestication

About 150 years ago, Darwin first introduced the concept of domestication syndrome, that domestic animals tended to share some common characteristics, such as more docility and frequent estrus cycles (Darwin, 2010; Wilkins et al., 2014). The domestication genes that cause genetic differentiation between wild and domestic populations appear in the early stages of domestication (Larson et al., 2014). We compared the diversity of CNVs between RJF and six domestic chicken populations to identify genes that may further illuminate the genetic basis of chicken domestication. A total of 255 protein-coding genes, predominantly involved in the development of nervous, tissue and reproductive system, were detected.

Previous reports demonstrated that neuronal development and behavior modification are under strong selection during long-term domestication progresses (Albert et al., 2008; Sanchez-Villagra et al., 2016; Zhang et al., 2018). More than 15 genes associated with the development and function of the neural system exhibited copy number variations between domestic breeds and RJF. Seven out of these genes were differentially expressed in cerebrum between domestic breeds and RJF. Except for *ANKRD11*, *NRG3*, and *MYT1*, the other genes were detected copy number variation for the first time. *NRG3* showed high copy number deletion in domestic chickens, which is consistent with previous study (Abe et al., 2017). As mentioned, *NRG3* has been implicated in severe neurological disorders with developmental origins (Meier et al., 2013; Li et al., 2020). *ANKRD11* showed copy number deletion in people with autism spectrum disorder compared to healthy persons (Marshall et al., 2008). The knockdown of *ANKRD11* exhibited markedly reduced neurons dendrite outgrowth (Ka and Kim, 2018). The overexpression of *MYT1* was suggested to help reduce anxiety in rats and has been associated with intellectual disability previously (Bahi and Dreyer, 2017). *BICD1*, *UNK*, and *KIF3C* are essential for neuron differentiation during development. *BICD1*, a conserved gene in *Drosophila*, *C. elegans* (Aguirre-Chen et al., 2011), and human (Baens and Marynen, 1997), is highly expressed in the developing central and peripheral nervous systems and plays an important role in neuronal homeostasis as a regulator of neurotrophin signaling (Terenzio et al., 2014). Loss of *UNK* alters the number of neural stem cells and neural progenitors resulting in increased neurogenesis (Maierbrugger et al., 2020). *KIF3C*, one of the kinesin-related motor subunits of the *KIF3* family, is selectively expressed in the nervous system during embryonic development (Navone et al., 2001). *FBXO41* and *EPS15L1* are associated with the defects in central nervous system (Mukherjee et al., 2015; King et al., 2019; Milesi et al., 2019). *SEMA3F*, the class 3 subfamily of semaphorins, was found to contribute to the development of neuronal circuitry related to anxiety and fear responses in *SEMA3F*-knockout mice (Milesi et al., 2019). Besides, the transcription factors of *nuclear receptor TLX* (*NR2E1*) might be regarded as a key regulator of neural development and differentiation (Matsushita et al., 2014; Sun et al., 2017).

The domestication of wild animals is a process of human civilization and food production. Therefore, improving production performances is an important part for chicken domestication. More than nine genes correlated with energy metabolism, reproduction function or cell growth and proliferation were screened. Three representative genes, *GBE1*, *HEP21*, and *INHA*, were first found to have copy number variation between domesticated and wild chickens. *GBE1* is involved in the metabolism of carbohydrate, presenting one copy deletion in all six domesticated chicken breeds. This gene was reported to be related with abdominal fat traits and presented higher expression levels in fast-growing chickens than in slow-growing chickens (Claire D'andre et al., 2013) or in fat chicken line than in lean chickens (Jin et al., 2017). *HEP21*, a gene unique to poultry, has doubled copy number variation in domesticated breeds. The expression of *HEP21* was associated with the development and function of chicken oviduct (Lim and Song, 2014), and SNPs in the *HEP21* gene have been reported to be related with sexual maturity (Chen et al., 2020). *INHA* protein is secreted by the avian granulosa cells of preovulatory follicles, and its gene expression level changed with the follicle development (Cui et al., 2019).

Improving disease resistance and immunocompetence of livestock is a precondition for security and abundance of food in the domestication of animals. At least ten genes were found to participate in the pathways of inflammatory and immune response. *CBFA2T3*, a factor in human acute myeloid leukemia, promotes myeloid differentiation (Steinauer et al., 2020). In cattle, this gene is found under selection pressure, which might be related to physiological adaptations to the environment and disease resistance (Ben-Jemaa et al., 2020). *IL22RA1* is one of the receptors for IL-22, an important cytokine involved in host defense and inflammatory responses (Gaudino et al., 2020). The expression of *ARIH2* is significantly altered in chicken immune organs after vaccine immunization (Wu et al., 2019). The biological function of *TNFAIP8* has been extensively investigated and was reported to play vital roles in modulating inflammation and immunity (Lou and Liu, 2011). *GATA3* was shown to regulate the differentiation and proliferation of T-cell (Ho et al., 2009). And *HERPUD1* is found differentially expressed in chickens under stress (Guo Y. et al., 2020).

### Breed-Specific Genes

Obviously, CNV plays a crucial role in chicken domestication and provides us a new perspective for studying the genetic mechanism underlying complex phenotypes. Therefore, copy number information exclusive to each domestic breed was screened to explore the genes contributing to breed characteristics. Some genes were discovered by previous study, such as *EDN3*, *SOCS2*, *HOXB7*, and *HOXB8*, but most genes relating to breed characteristics were identified for the first time. Among the discovered genes, some are already verified contributing to appearance, growth or reproduction traits in chicken, and the others, whose biological function are discussed based on studies in human or other animals, are worthy of further researches.

For RW chickens, the significantly high copy number duplications of *POLR3H*, *MCM9*, *DOCK3*, *AKR1B1L*, *ASF1A*, *ATP2B4*, and *MYH1B* may contribute to the fast-growing traits. Knocking down any of the three genes, that is, *POLR3H*, *MCM9*, and *DOCK3*, would cause delayed development or short growth (Lutzmann et al., 2012; Qin et al., 2015; Iwata-Otsubo et al., 2018; Franca et al., 2019; Guo T. et al., 2020). *AKR1B1L*, a member of the aldo/keto reductase superfamily, was related to body length and fat deposition in cattle (An et al., 2020) and chickens (Claire D'andre et al., 2013). *ASF1A* was reported to be a candidate gene for muscle weight and meat quality (Liu et al., 2013; Zhang et al., 2020). *ATP2B4* is one of ATPase family that regulates intracellular calcium homeostasis (De Koning et al., 2001) and plays an important role in bone development (Wu et al., 2004; Kim et al., 2012). Furthermore, *MYH1B* was also demonstrated to participate in the early development of breast muscle or regeneration of muscle fibers in broilers (Pampouille et al., 2018).

For WL chickens, the copy number deletion of *CSMD1* and *NTRK3* may be involved in the outstanding reproduction traits, because *NTRK3* and *CSMD1* have been reported to play crucial roles in follicle development, ovarian quality, and infertility in human and chicken (Lachman et al., 2007; Nilsson et al., 2009; Lee et al., 2019).

In SILK chickens, which are famous for their black skin and bone, four genes (*EDN3*, *SLMO2*, *TUBB1*, and *GFPT1*) exhibited a significant copy number increase, which may be responsible for melanin formation and deposition. *EDN3*, a well-known candidate for melanocytic proliferation and maintenance, has been reported to be strongly related to dermal pigmentation in SILK chickens (Shinomiya et al., 2012). *SLMO2* and *TUBB1* have also been discovered with around 4 and 5 copy number duplications, respectively, and they showed significantly high expression in both skin and muscle tissues in SILK chickens (Dorshorst et al., 2011). *GFPT1* mutation has been reported to specifically affect the production of melanin in zebrafish (Yang et al., 2007).

LXG chickens, a typical cockfighting breed characterized by aggressiveness, strong bone strength, and powerful muscle, exclusively exhibited duplications on *SOCS2*, *PRDM5*, *CRADD*, and *SORCS2*. *SOCS2* (Lorentzon et al., 2005) and *PRDM5* (Galli et al., 2012) may contribute to the high bone mineral density. And the increased copy number and gene expression variation of *SOCS2* in gamecocks were validated by Bi et al. (2017). The one more copy number increase of *CRADD* in LXG may be responsible for fast muscle growth trait (Horvat and Medrano, 1998; Smith et al., 2000). Meanwhile, a CNV region (GGA: 80,834,764–80,851,024 bp) overlapping the gene *SORCS2* was particularly detected in LXG. *SORCS2* was suggested to be a candidate gene for the aggressive behavior of LXG, because knockdown of *SORCS2* would significantly decrease the expression of dopamine receptor genes and nerve growth factor (Li et al., 2016).

YOU chicken is a Chinese native breed with special appearance characteristics, including crests, beards, feathered legs, and polydactyly. Recently, Yang et al. (2020) deciphered that the copy number variation in *HOXB7* and *HOXB8* is responsible for the beard trait, which coincides exactly with the CNV detected in our study, especially exhibiting two copy number increase in YOU chicken.

## Conclusion

This study provides genome-wide CNV information in one wild chicken breed (RJF), two commercial breeds, and four Chinese local breeds to synthetically understand the process of chicken domestication and to exclusively identify the genes contributing to breed characteristics. A total of 11,123 CNVRs with 19,329 duplications and 98,736 deletions were detected, covering 7% of the autosome genome and overlapping 2,636 protein-coding genes. The PCA results revealed that CNV is a powerful method for exploring evolution and domestication. In chicken evolution, genes related to nervous system, sensory, and follicle development were supposed to have pivotal roles. Meanwhile, cognitive and behavior changes, reproduction function, metabolism, and immunity are under strong selection during long-term animal domestication process. What’s more, some valuable genes contributing to fast growth, high reproduction, and distinct breed characteristics were also identified in this paper, which deserve further investigations. Taken together, the present study provides numerous copy number information for chickens and it is a valuable resource to facilitate genetic and functional investigation of domestication and economic traits in chickens.

## Data Availability

The original contributions presented in the study are included in the article/Supplementary Material, further inquiries can be directed to the corresponding authors.

## Abbreviations

CNV, Copy number variation; CNVR, Copy number variation region; DEGs, Differentially expressed genes; GO, Gene ontology; IPA, Ingenuity pathways analysis; KEGG, Kyoto encyclopedia of genes and genomes; LXG, Luxi game; PCA, Principal component analysis; RJF, Red jungle fowl; RW, Recessive white rock; SILK, Silkie; SNP, Single nucleotide polymorphism; SRA, Sequence read archive; WL, White leghorn; XH, Xinghua; YOU, Beijing YOU.
