# Supplementary figures and images for: Population Genomic Sequencing Delineates Global Landscape of Copy Number Variations that Drive Domestication and Breed Formation of in Chicken
Source: Front Genet. 2022 Mar 22;13:830393. doi: 10.3389/fgene.2022.830393 (PMC8980806; doi:10.3389/fgene.2022.830393)

NRG3 chr6:2,926,344-3,201,956

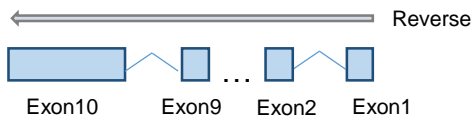

GBE1 chr1:94,474,405-94,635.350

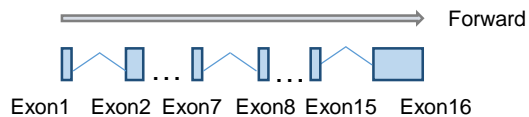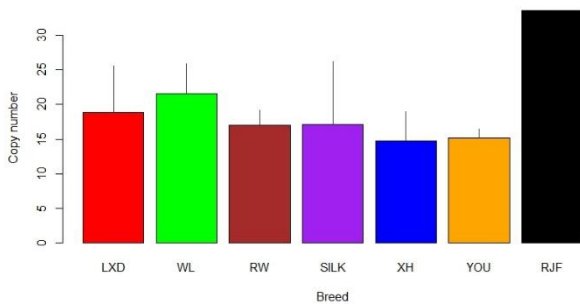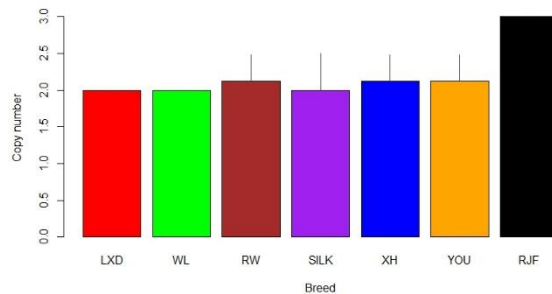

Supplement: Supplementary file 1 [file DataSheet2.PDF]

A

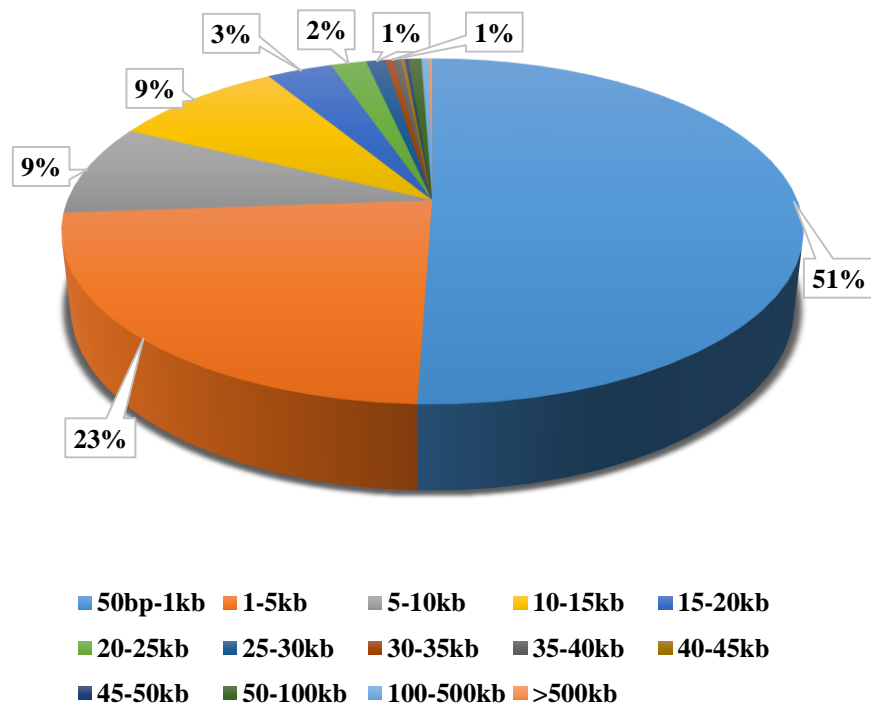

B

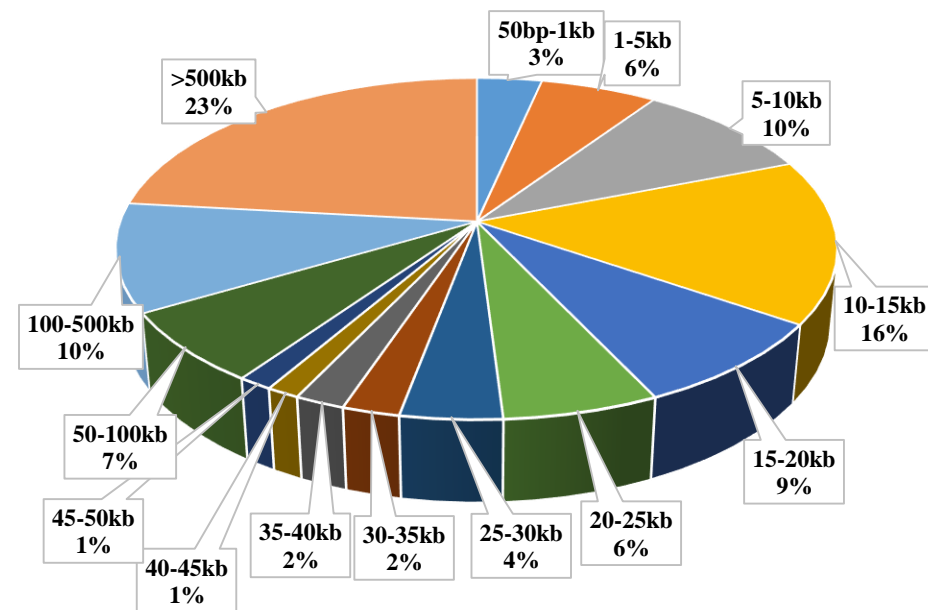

Supplement: Supplementary file 3 [file DataSheet1.PDF]
